# Carcinoma of the Breast in Nigeria

**DOI:** 10.1038/bjc.1963.74

**Published:** 1963-12

**Authors:** J. B. Pearson

## Abstract

**Images:**


### BRITISH JOURNAL OF CANCER

VOL. XVII DECEMBER, 1963 NO. 4

### CARCINOMA OF THE BREAST IN NIGERIA
### A Review of 100 Patients

J. B. PEARSON

*From the Department of Surgery, University College Hospital, Ibadan, Nigeria.*

Received for publication August 12, 1963

WHILE this disease has been well documented in Europe and America, no such data exist for Nigeria. This study has been done in order to sketch in one part of the pathological map of West Africa, which is at present almost blank, and also to enable undergraduate teaching to be modified if need be.

### MATERIAL

Although most of our patients came from the Western Region of Nigeria within 100 miles of Ibadan, yet some travelled over 500 miles within the Federation to seek medical help. From 1957 (January) to 1963 (June), 100 patients were admitted to University College Hospital with carcinoma of the breast. This is a retrospective study of these patients, one-quarter of whom have been examined by the author.

*Relative frequency*

Carcinoma of the breast comprises 6·7 per cent of all tumours and 12·4 per cent of female carcinomas seen at the Ibadan Cancer Registry (Maclean and Edington, 1963, personal communication). The comparable figure for Uganda, French West Africa and Ghana is respectively 4 per cent, 5·4 per cent and 5·4 per cent of all tumours (Davies and Wilson, 1954 ; Camain, 1954 ; Edington, 1956). In the Ibadan Register it is the fourth commonest tumour in females,tumours of reticulo-endothelial system, uterine cervix, and Burkitt's tumour taking precedence. At the University Hospital, on an average, there were 2300 surgical admissions each year, 14 (0·5 per cent) being for breast cancer. This contrasts with 1·6 per cent admissions to surgical wards at the University College Hospital in the West Indies which serves a negro population (Annamunthodo, 1958).

*Age*

The average age of patients with breast cancer in Nigeria is 44·9 years, compared with 55·3 years in England (Annual Cancer Report, United Birmingham Hospitals, 1957). From a study of the age incidence chart (Fig. 1) it is calculated that 74 per cent patients are in the 35 to 64 age group, with a peak incidence in the quinquennium 40 to 44. This corresponds to the findings in Japan (Grady, 1955), and contrasts with most countries, including the West Indies and America, where 80 per cent are in the 35 to 64 age group, and the peak incidence for the disease is 45 to 49 years. (Annamunthodo, 1958 ; Nathanson and Welch, 1936). Forty-nine patients were premenopausal, and 51 were post-menopausal.

*Fertility*

Of the 78 patients for whom an obstetric history was available, five had no children, one was a man, and the number of children born to the remaining 72 is shown in the Parity table (Fig. 2).

FIG. 1.—Age incidence of 100 cases of carcinoma of the breast.

*Breast Feeding*

During the reproductive life of Nigerian women, the breast is in a state of almost continuous activity. Weaning is governed by the occurrence of a further pregnancy, and indeed good nutrition in the early years of life is dependent upon maintenance of breast feeding for longer than in the western world. In extreme cases a child may be breast fed for up to three years, while eighteen months is not uncommon, with the result that one sometimes sees two children of different ages being breast fed at the same time.

### CLINICAL FEATURES

The cardinal symptoms and signs are recorded (Table I).

TABLE I.—*Clinical Features*

| | | Nipple signs | | |
|---|---|---|---|---|
| Lump | Pain | Level | Retraction | Discharge |
| 100% | Present 74% | Elevated 27% | 26% | 18% { 9 lactating |
| Breast alone : 89% | Absent 21% | Level 11% | | 4 blood |
| Breast and axilla : 8% | No record 5% | Depressed 10% | | 3 sero-sanguinous |
| Axilla alone : 3% | | Destroyed 9% | | 2 pus |
| — | — | No record 43% | — | — |

*Tumour*

The length of history (Fig. 3) is shown for 95 patients, the remaining five being doubtful. It should be emphasized, however, that in a country with far too few hospitals and a tradition of priority being given to acute cases, brevity of history as related by the patient may be designed to obtain urgent admission.

The maximum measured diameter of 63 tumours (Fig. 4) is shown. The remaining 37 patients were excluded because the tumour had been described as "extensive" without measurement.

FIG. 2.—Parity of 77 patients with carcinoma of the breast.

In 80 per cent of the patients a spot diagnosis was all too easy, as is exemplified in the photographs (Fig. 5).

*Anatomical location*

The right breast was affected in 54 patients, and the left in 43. Both were involved in 3 cases. This predominance of tumours in the right breast is more pronounced than in the West Indies (Annamunthodo, 1958). Reports from other countries show that the left breast is more often affected (Grady, 1955 ; Berkson *et al.*, 1957 ; Annual Cancer Report, United Birmingham Hospitals, 1957).

The incidence of carcinoma in different quadrants of the breast is shown (Table II). It will be noted that in other series (Annamunthodo, 1958 ; Alrich, Liddle and Morton, 1957 ; Marshall and Higginbotham, 1938) while 50 per cent to 60 per cent of tumours were in the upper outer quadrant, in the present series only 22 per cent tumours were in this site, which is still the commonest quadrant. This is explained by the larger number of our patients (42 per cent) in which the whole breast or more than one quadrant was involved.

FIG. 3.—Length of history of 95 patients with carcinoma of the breast.

FIG. 4.—Size of tumour in 63 patients with carcinoma of the breast.

### EXPLANATION OF PLATE.

FIG. 5.—Typical appearances of carcinoma of the breast in Nigeria.

5

TABLE II.—*Anatomical Location of the Breast Carcinoma and Nodes Involved for Each Quadrant*

| | Ibadan, Nigeria | | Virginia, U.S.A. | | |
|---|---|---|---|---|---|
| Location | Number | Number with involved axillary nodes | % | Percentage with involved axillary nodes | West Indies % |
| Upper outer quadrant | 22 | 18 (81·8%) | 48 | 58 | 50·8 |
| Upper inner quadrant | 12 | 9 (75%) | 15 | 48 | 11·9 |
| Lower inner quadrant | 0 | 0 (0%) | 6 | 40 | 3·2 |
| Lower outer quadrant | 3 | 2 (66·6%) | 9 | 41 | 0·4 |
| Whole breast | 30 | 27 (95%) | 8 | 75 | 16·7 |
| Retro-areolar | 15 | 13 (86·6%) | 13 | 52 | 2·4 |
| Both upper quadrants | 11 | 9 (81·8%) | — | — | — |
| Both inner quadrants | 5 | 3 (60%) | — | — | — |
| Both outer quadrants | 1 | 1 (100%) | — | — | — |
| Not noted | 1 | — | — | — | 1·6 |
| Other (Paget's disease, both breasts, etc.) | 0 | 0 | — | — | 9·6 |

It is suggested that the classical quadrant method of description is unsuitable for most Nigerian breasts. Breast shape was noted in 65 patients of which 46 (70 per cent) had pendulous breasts, the nipple being the most dependant part. This explains the small number of patients with involvement of the lower quadrants (9 per cent), for in most there is little tissue from which lower quadrant growths can arise.

*The extent of the growth*

This is summarized in Table III.

TABLE III.—*The Extent of the Growth*

| Skin involvement | | Fixation to pectoralis major or chest wall | Lymphatic spread | Haematogenous spread |
|---|---|---|---|---|
| Tethering | 92 | | 83 Hard axillary nodes (28 fixed ; 9 bilateral) | 11 Pulmonary metastases |
| Peau d'orange | 55 | | | — |
| Ulcer | 41 | 38 | 15 Supraclavicular nodes | 6 Skeletal metastases |
| Cancer en cuirasse | 13 | | 9 Oedema of arm | — |
| Inflammation | 11 | | — | — |

The tumours were staged according to the well known Manchester Classification :

Stage I : Primary tumour is mobile. Skin involvement, if present, is not wide of the tumour—One case only.

Stage II : As Stage I, but there are palpable lymph nodes in the axilla of the same side—Four cases.

Stage III : Tumour has skin involved wide of it, and may be fixed to underlying muscles, though not the chest wall—40 cases.

Stage IV : Growth has extended beyond the breast area (fixed to chest wall, fixed axillary nodes, distant metastasis)—55 cases.

The staging of growths in Nigeria, England and the West Indies is contrasted (Table IV). It will be seen that the majority of the Nigerian series are beyond the limits of curative surgery.

TABLE IV.—*Comparison of Staging of Growths in Ibadan, West Indies and Birmingham*

| | Ibadan | West Indies | Birmingham |
|---|---|---|---|
| Stage I | 1 | 18 (14·3%) | 26·8% |
| Stage II | 4 | 65 (51·6%) | 21·4% |
| Stage III | 40 | 18 (14·3%) | 26·2% |
| Stage IV | 55 | 25 (19·8%) | 25·7% |
| Total | 100 | 126 (100%) | 100% (4724) |

*Histology of the growths*

The growths were placed in one of three grades, according to the method described by Bloom (1950) and Bloom and Richardson (1957), based on the principles laid down by Greenough (Greenough, 1925). None of the tumours fell into Grade I, that of low malignancy. Twenty per cent were Grade II (moderate malignancy) and 80 per cent were Grade III (highly malignant) (Edington, 1963, personal communication). This compares unfavourably with Bloom and Richardson's, 1544 patients, 26 per cent of which were Grade I, 45 per cent Grade II and 29 per cent Grade III.

### CONCLUSIONS

1. Carcinoma of the breast in Nigeria presents a picture of growths as rampant as the vegetation of the tropical rain forest.

2. There is a variety of reasons for growths being more advanced by the time patients reach hospital. Lack of transport, hospitals and doctors, coupled with the present subordinate status of women in a society that enforces continuous care of the family so long as the woman is able to work, all contribute to late attendance. Anxiety and fear almost invariably cause the patient to subject herself to the abortive ministrations of nature doctors before she seeks scientific medical help. In addition to these factors, we have shown that the tumours are more highly malignant.

3. The patients seen are younger than in Europe or America.

4. There was only one case of carcinoma of a male breast. This incidence (1 per cent) agrees with that in America and England, and is lower than the 10 per cent reported in Kampala (Knowelden, 1957) and the 4 per cent in Johannesburg (Higginson and Oettlé, 1947). However, the male percentage in Kampala and Johannesburg failed to take into account the much lower incidence of female breast cancer in these places (6·8 per 100,000, compared with 43·6 per 100,000 in American whites—Davies, 1963, personal communication).

### SUMMARY

1. One hundred patients with carcinoma of the breast attending the University Hospital at Ibadan between 1957 and 1963 are reviewed.

2. The histories, clinical features and histology are described.

3. The growths appear approximately a decade earlier than in Europe, are much more widespread, and belong to a more malignant histological grade.

My thanks are due to Prof. W. W. Davey for much helpful criticism of this paper and Prof. G. M. Edington for personally reviewing the histology of the tumours.

### REFERENCES

ALRICH, E. M., LIDDLE, H. V. AND MORTON, C. B.—(1957) *Ann. Surg.*, **145**, 799.
ANNAMUNTHODO, H.—(1958) *W. Ind. med. J.*, **7**, 93.
Annual Cancer Report, United Birmingham Hospitals—(1957) **3**, 5.
BERKSON, J., HARRINGTON, S. W., CLAGETT, O. T., KIRKLIN, J. W., DOCKERTY, M. B. AND McDONALD, J. R.—(1957) *Proc. Mayo Clin.* **32**, 645.
BLOOM, H. J. G.—(1950) *Brit. J. Cancer*, **4**, 259.
*Idem* AND RICHARDSON, W. W.—(1957) *Ibid.*, **11**, 359.
CAMAIN, R.—(1954) *Bull. Soc. Pat. exot.* **47**, 614.
DAVIES, J. N. P. AND WILSON, B. A.—(1954) *E. Afr. med. J.*, **31**, 396.
EDINGTON, G. M.—(1956) *Brit. J. Cancer*, **10**, 595.
GRADY, H. G.—(1955) 'Trans. 5th meet. Int. Soc. Geogr. Path'. Basel (S. Karger), p. 685.
GREENOUGH, R. B.—(1925) *J. Cancer Res.*, **9**, 453.
HIGGINSON, J. and OETTLÉ, A. G.—(1957) *Acta. Un. int. Cancr.*, **13**, 949.
KNOWELDEN, J.—(1957) *Proc. R. Soc. Med.*, **50**, 249.
MARSHALL, S. F. AND HIGGINBOTHAM, J.—(1938) *Surg. Clin. N. Amer.* **18**, 615.
NATHANSON, I. T. AND WELCH, C. E.—(1936) *Amer. J. Cancer*, **28**, 35.


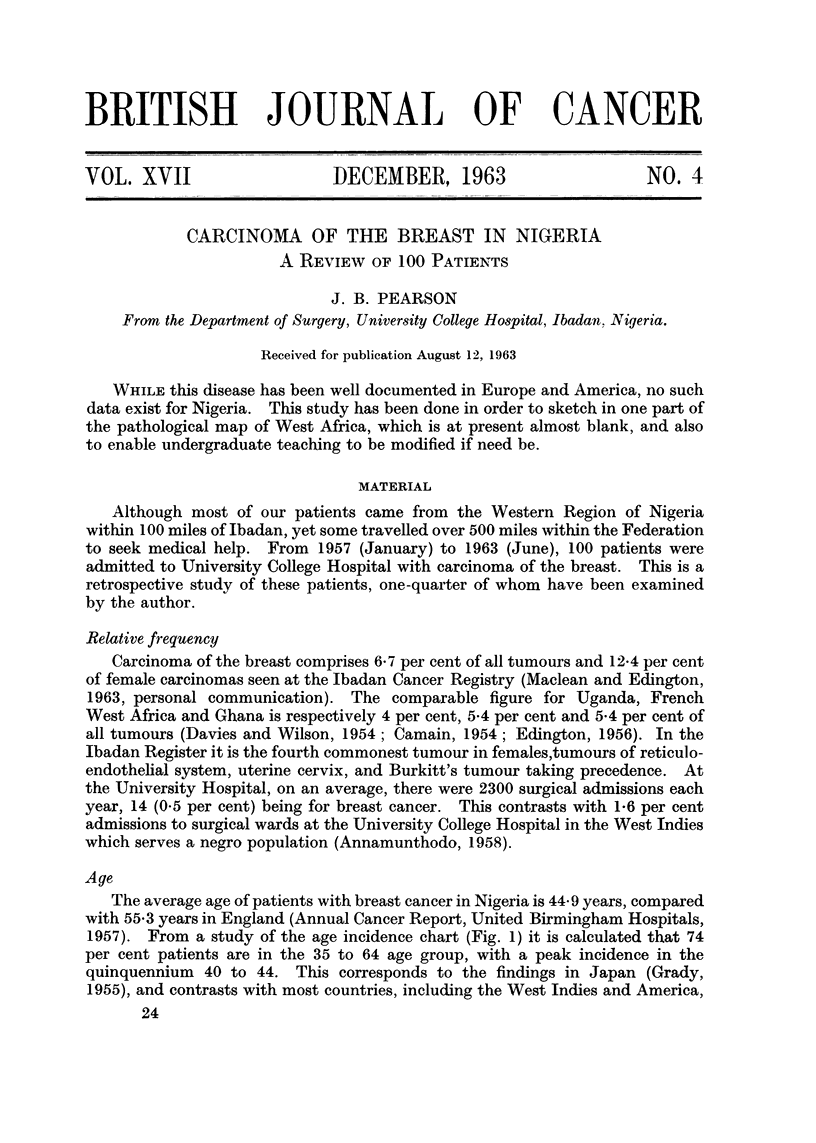

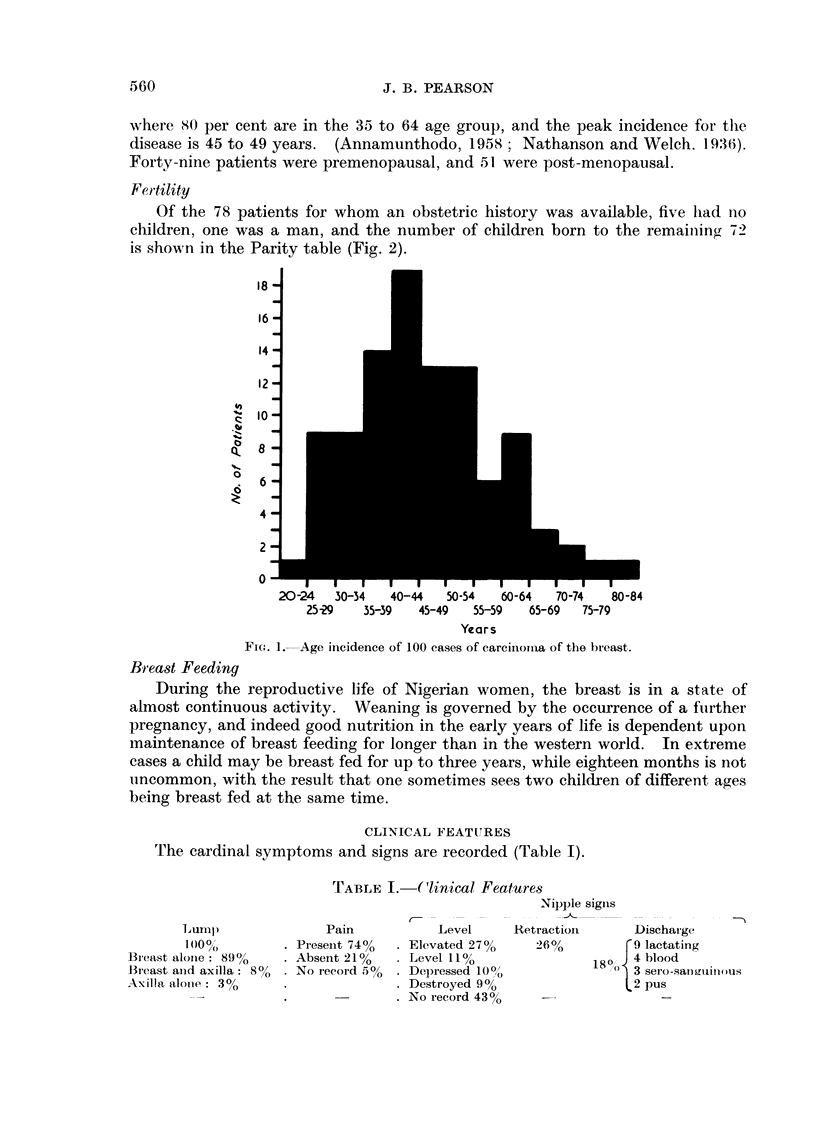

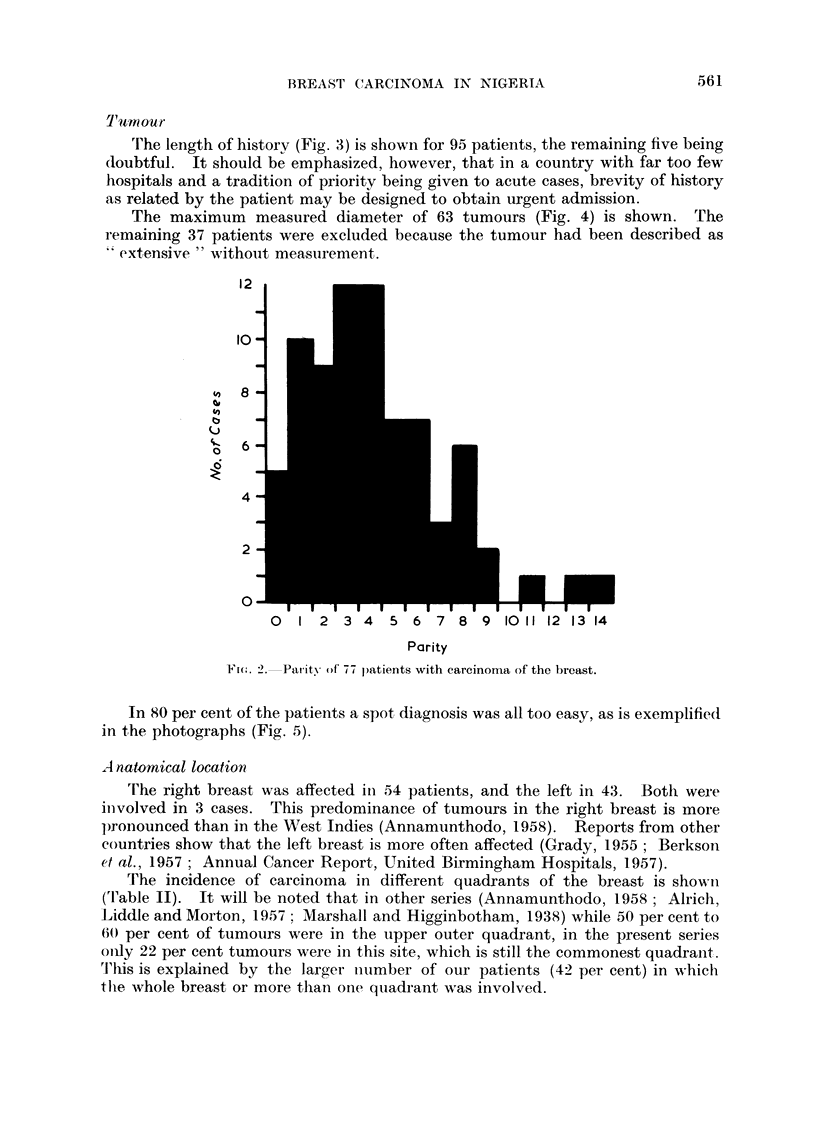

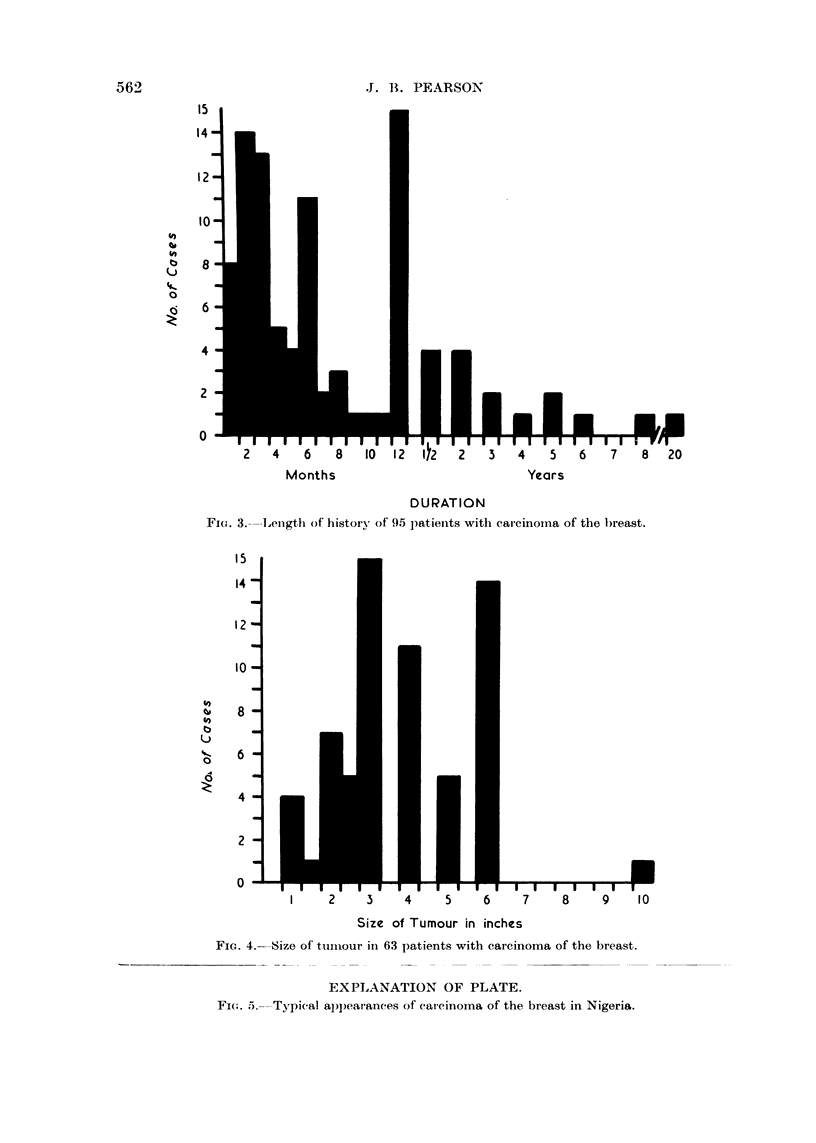

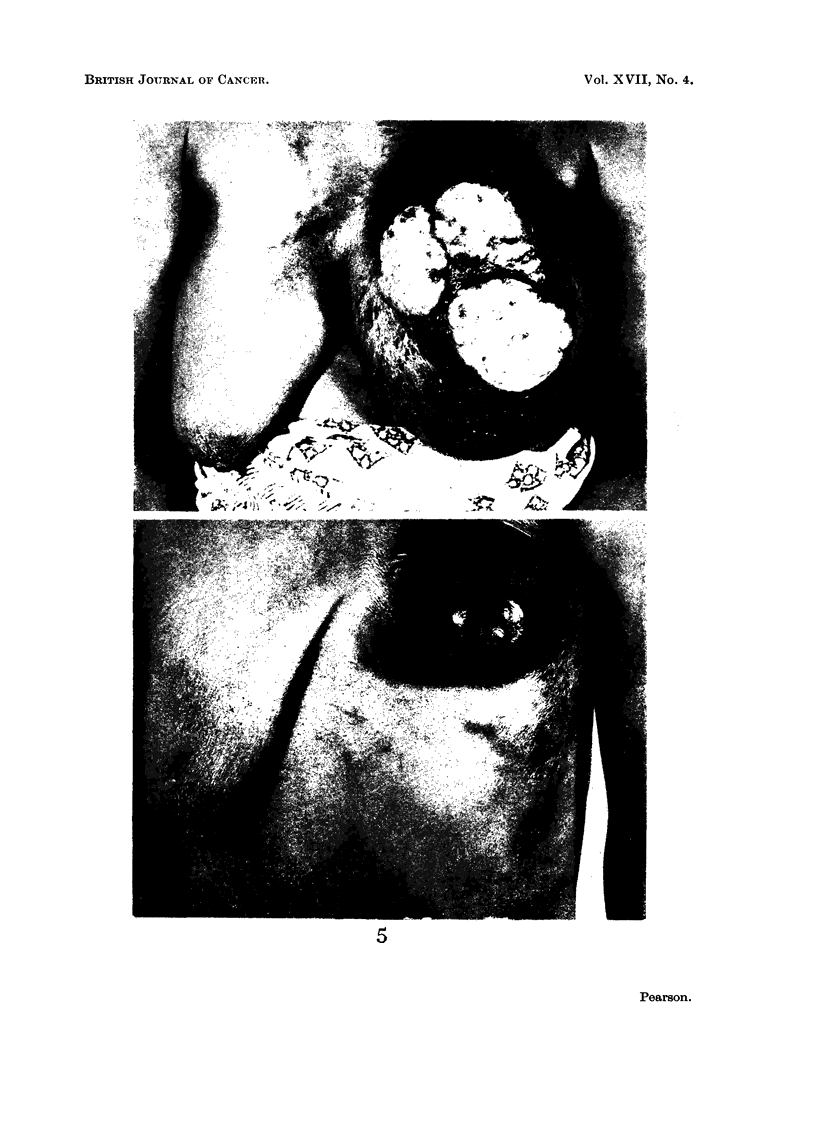

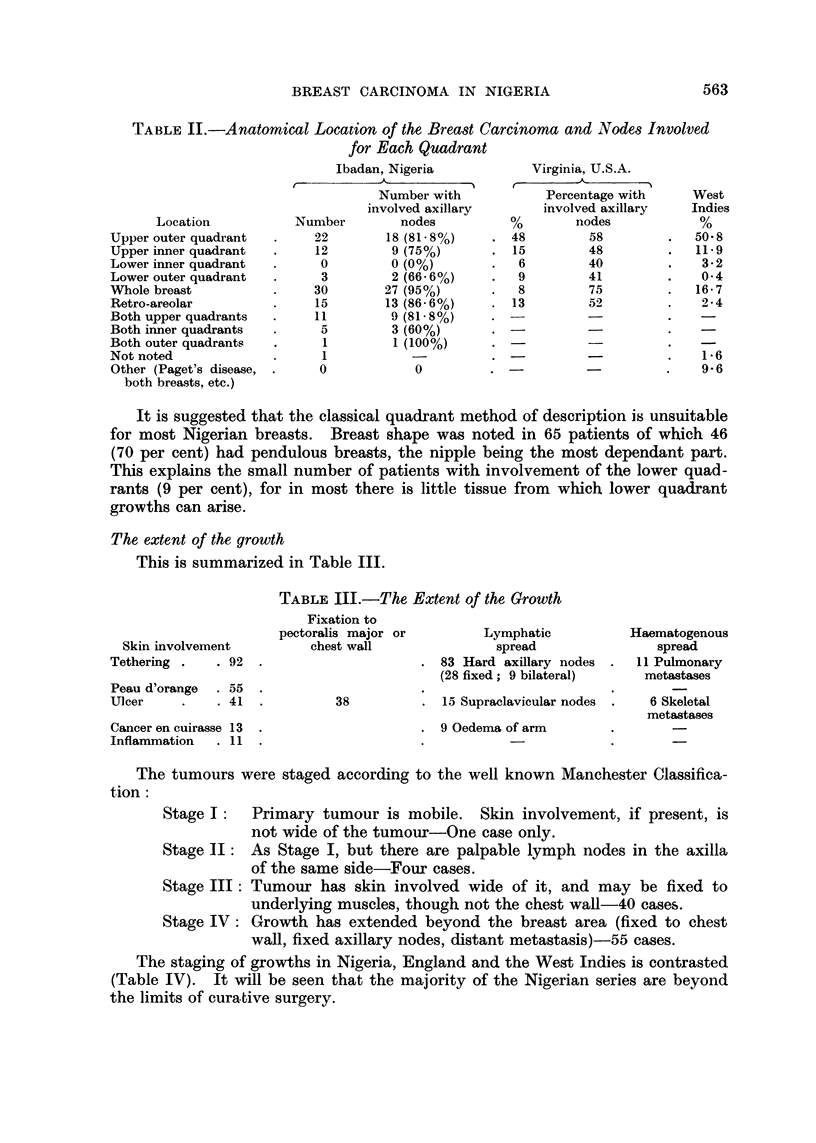

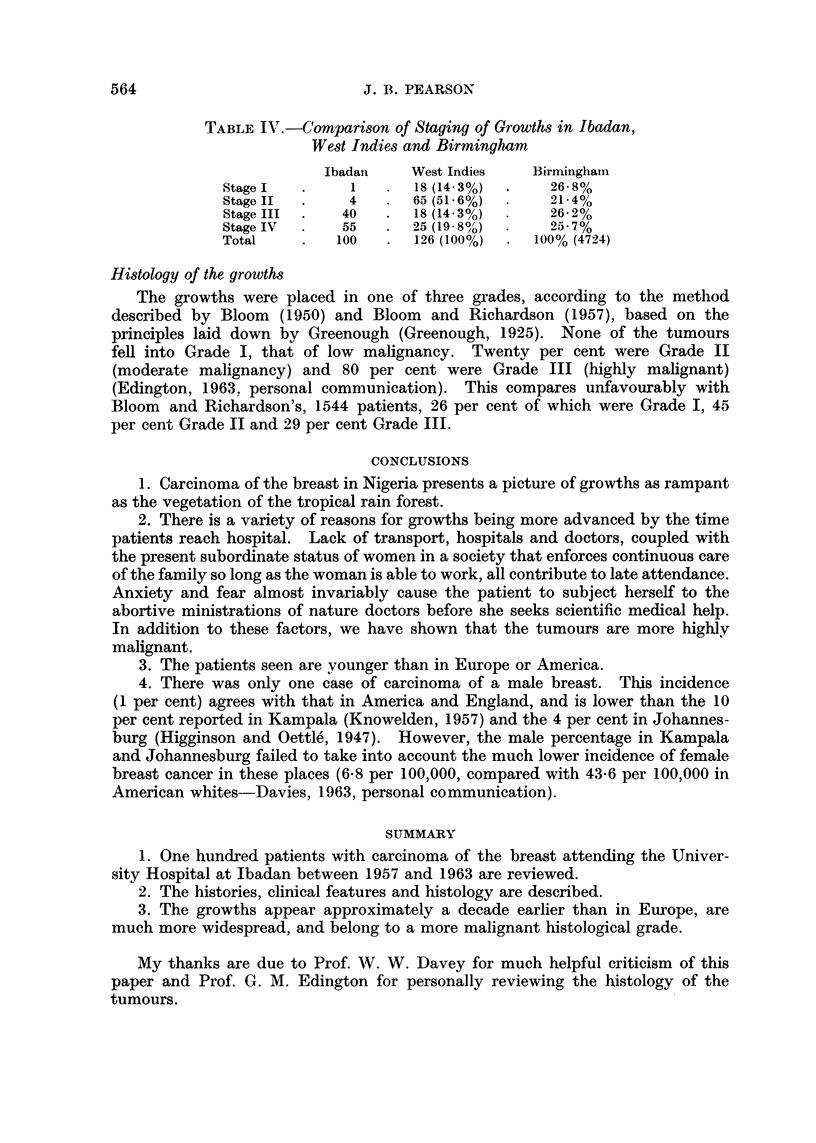

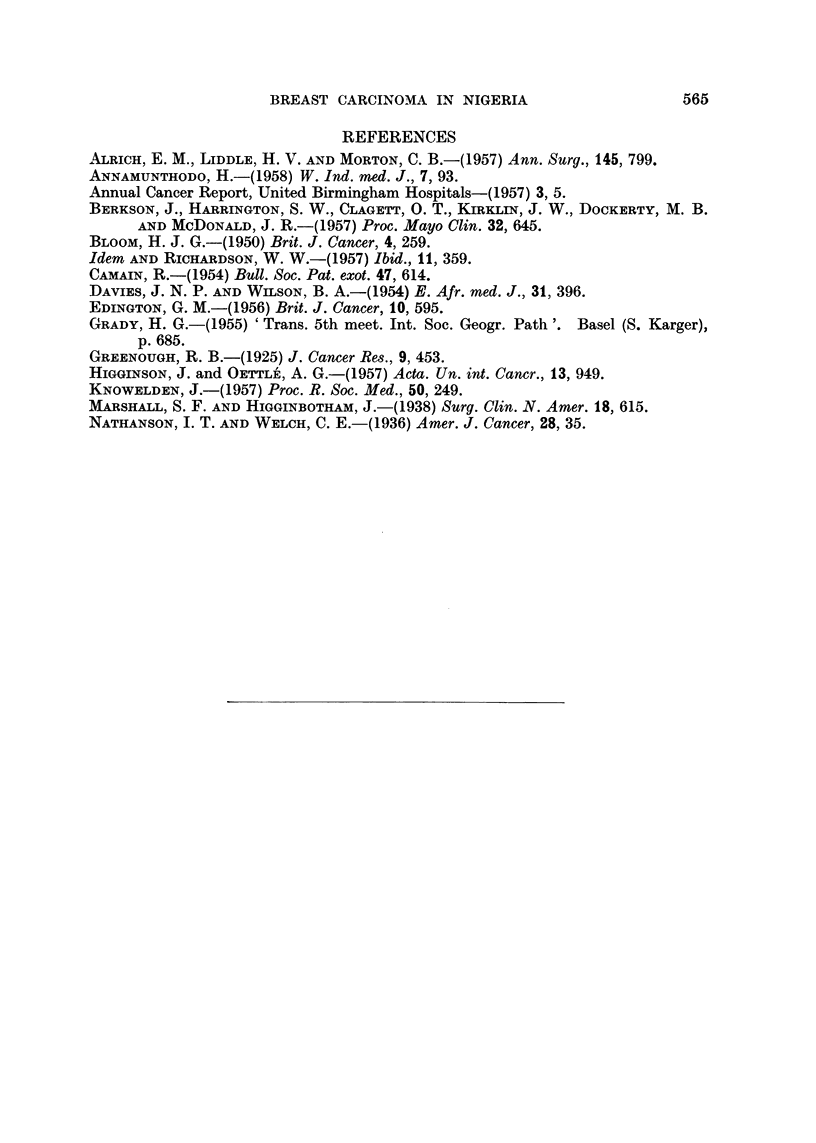


## References

[OCR_00523] ALRICH E. M., LIDDLE H. V., MORTON C. B. (1957). Carcinoma of the breast: results of surgical treatment; some anatomic and endocrine considerations.. Ann Surg.

[OCR_00530] BERKSON J., HARRINGTON S. W., CLAGETT O. T., KIRKLIN J. W., DOCKERTY M. B., McDONALD J. R. (1957). Mortality and survival in surgically treated cancer of the breast: a statistical summary of some experience of the Mayo Clinic.. Proc Staff Meet Mayo Clin.

[OCR_00536] CAMAIN R. (1954). Aperçus sur le cancer en A.O.F.. Bull Soc Pathol Exot Filiales.

[OCR_00537] EDINGTON G. M. (1956). Malignant disease in the Gold Coast.. Br J Cancer.

[OCR_00545] HIGGINSON J., OETTLE A. G. (1957). The incidence of cancer in the South African Bantu.. Acta Unio Int Contra Cancrum.

[OCR_00546] KNOWELDEN J. (1957). Cancer in Kampala.. Proc R Soc Med.

